# Shikonin reduces endometriosis by inhibiting RANTES secretion and mononuclear macrophage chemotaxis

**DOI:** 10.3892/etm.2013.1458

**Published:** 2013-12-23

**Authors:** DONG-PING YUAN, LIN GU, JUN LONG, JIE CHEN, JIE NI, NING QIAN, YING-LI SHI

**Affiliations:** 1 State Key Laboratory of Reproductive Medicine, Department of Gynecology, Nanjing Maternity and Child Health Care Hospital, Nanjing Medical University, Nanjing, Jiangsu 210004, P.R. China; 2 Nanjing University of Chinese Medicine, Nanjing, Jiangsu 210004, P.R. China

**Keywords:** endometriosis, shikonin, RANTES, chemotaxis

## Abstract

Endometriosis is a common disease in females of reproductive age and has the classic characteristic of mononuclear cell infiltration into lesions. Shikonin is an anti-inflammatory phytocompound obtained from *Lithospermum erythrorhizon* whose potential therapeutic effects in the treatment of endometriosis remain unclear. The working hypothesis of the present study was that shikonin is capable of inhibiting the development of endometriosis by inhibiting the chemotactic effect. In a murine model of endometriosis, shikonin significantly inhibited the growth of human endometrial tissue implanted into severe combined immunodeficiency (SCID) mice (P<0.05) and no adverse effects were observed. Mouse regulated upon activation normal T-cell expressed and secreted (mRANTES) levels in the peritoneal fluid of the animal endometriosis model were higher than those in normal SCID mice (P<0.05) and decreased significantly following shikonin treatment in a dose-dependent manner (P<0.05). Peritoneal fluid from SCID mice treated with shikonin inhibited the chemotaxis of monocytes; this inhibitory effect was eradicated by mRANTES antibody. *In vitro*, shikonin significantly inhibited RANTES expression in U937 cells that were cultured alone or co-cultured with human mesothelial and endometrial stromal cells. In addition, shikonin inhibited the RANTES-induced chemotaxis of U937 cells (P<0.05). The results indicate that shikonin inhibits the development of endometriosis by various mechanisms, including the inhibition of RANTES expression and the reduction of mononuclear cell migration to lesions. Therefore, shikonin may be a novel, useful and safe agent for treating endometriosis.

## Introduction

Endometriosis is a gynecological complication characterized by extra-uterine localization of endometrial tissue, mainly in pelvic organs. The disease affects 5–10% of females of reproductive age (1). Clinical presentations of endometriosis include persistent worsening of pelvic adhesions, pain and infertility. Little is known about its etiopathology. With the exception of the symptomatic treatment of endometriosis-associated pain, only two main suboptimal therapeutic approaches (hormonal and invasive surgery) are generally recommended to patients (2). Thus, it is important to identify a new specific target for the treatment of endometriosis.

Shikonin is the main active component of *Lithopermum erythrorhizon*, a perennial herb in the Boraginaceae family. Shikonin is a naphthoquinone compound (molecular formula, C_16_H_16_O_5_; molecular weight, 288) that has anti-inflammatory, antitumor and immunomodulatory bioactivities (3–5). In a previous study in which the effects of shikonin against human immunodeficiency virus were investigated, shikonin antagonized chemokine function by downregulating the expression of chemokine receptors, including CCR5, indicating that this compound is a possible pan-chemokine inhibitor (6).

Previously, ectopic and normal endometrial tissues were screened for 18 chemokine receptors (CCR1-10, CXCR1-6, XCR1 and CX3CR1) and it was found that CCR5 expression levels were higher in the ectopic focus than in the eutopic endometrium in endometriosis patients. Regulated upon activation normal T-cell expressed and secreted (RANTES) is the ligand for CCR5, and it has been confirmed that the chemokine and its receptors are bioactive in endometriosis (7–9). These observations indicate that shikonin may be a promising endometriosis treatment. In the present study, the potential therapeutic effects and mechanism of shikonin were assessed for the treatment of endometriosis.

## Materials and methods

### Reagents

Shikonin (purity, ≥97%) was provided by the National Institute for the Control of Pharmaceutical and Biological Products (Beijing, China) and was dissolved in DMSO. Other reagents included: triptorelin (Beaufour-Ipsen Pharmaceutical Co., Ltd., Tianjin, China); TRIzol reagent (Gibco-BRL, Gaithersburg, MD, USA); Real Time polymerase chain reaction (PCR) Core kit (Takara Bio, Inc., Dalian, China); carboxyfluorescein succinimidyl ester (CFSE) and recombinant human RANTES (rhRANTES; R&D Systems, Wiesbaden, Germany); mouse monocyte isolation buffer (Lengtonbio Biological Co., Shanghai, China); immunostaining kits and keratin and vimentin monoclonal antibodies (MaxVision™ HRP-Polymer anti-Mouse IHC kit; Fuzhou Maixin Biotechnology Development Co., Ltd., Fuzhou, China); and enzyme-linked immunosorbent assay (ELISA) kits (BioSource International, Camarillo, CA, USA). All other chemicals were of analytical reagent grade.

### Human endometrial samples

Informed consent was obtained prior to endometrial biopsy collection and the study was approved by the Nanjing Medical University Ethics Committee (Nanjing, China). The ten donors were premenopausal patients with endometriosis (aged 24–42 years), who underwent laparoscopy and uterine curettage at the Nanjing Maternal and Child Health Hospital Affiliated with Nanjing Medical University. Fresh endometrial tissue was collected in Dulbecco’s modified eagle medium (Gibco-BRL) and cut into 1–2-mm diameter fragments under sterile conditions.

### Animal endometriosis model

In total, 70 severe combined immunodeficiency (SCID) female mice weighing 20–25 g, at 6–8 weeks-old [Beijing Vital River Laboratory Animals Co., Beijing, China; certificate of quality no: SCXK (Jing) 2007–0001], were housed under a 12 h light-dark cycle with food and water available *ad libitum* and pathogen-free conditions. All studies were approved by the experimental animal committee of Nanjing Medical University. Human endometrial fragments were implanted into the peritoneal cavity and mice were intramuscularly injected with estradiol benzoate (30 μg/kg) one day following implantation, in order to maintain the growth of the implanted endometrium. After 14 days, 10 mice were euthanized by cervical dislocation and the implanted endometrial tissue in the peritoneal cavity was examined.

### Groups and treatments

Model animals were randomly divided into the following groups (n=12 per group): Negative control, oral DMSO treatment; positive control, subcutaneous treatment with 30 μg/kg tryptorelin (a gonadotropin-releasing hormone agonist); and low-, intermediate- and high-shikonin dose groups that were orally treated with shikonin at doses of 2.5, 5 and 10 mg/kg, respectively. All animals were treated daily for 28 days. At the end of the experiment, mice were sacrificed by cervical dislocation and blood samples were collected. Implanted endometrial lesions were dissected and frozen directly in liquid nitrogen for PCR or fixed in 10% formalin and embedded in paraffin for immunohistochemical analysis. Lesion volume (V) was calculated using the formula: V (mm^3^) = 0.52 × length × width × height.

### Assessment of toxicity and adverse effects

All animals were closely monitored for abnormal behavioral changes and symptoms, including marked temperature change, diarrhea, weight loss, fur discoloration, lethargy, irritation and convulsion during treatment. All observations were performed by investigators blinded to the treatment group assignments.

Blood samples were collected following the sacrifice of the mice. Serum levels of alanine aminotransferase (ALT), aspartate aminotransferase (AST), blood urea nitrogen (BUN), creatinine (Cr), lactate dehydrogenase (LDH), creatine kinase (CK) and total protein, albumin and globulin were tested by auto analyzer (HITACHI 7020; Hitachi, Tokyo, Japan).

### Cell culture and co-culture

To study the potential role of shikonin on RANTES expression in a pelvic environment of endometriosis, a cell co-culture system [U937 cells-human peritoneal mesothelial cells (HPMCs)-endometrial stromal cell (ESCs)] was constructed. The ESCs, HMrSV5 (HPMCs) and U937 human monocyte cell line (Cell Bank, Chinese Academy of Sciences, Shanghai, China) were cultured according to previously described methods (7).

The HPMCs were cultured in 12-well plates at a concentration of 1×10^5^ cells/well until they adhered to the plastic. The media were removed and the ESCs and U937 cells were spread over the HPMC monolayer at the same concentration. HPMC, ESC and U937 cells cultured alone in the same media were used as controls.

### Cell treatment

Cells were seeded on 12-well plates (1×10^5^/well) and the medium was changed to 2.5% fetal bovine serum (FBS) after 24 h. Cells were treated with various shikonin concentrations (range, 0.5–50 μM) for 24 h. Untreated cells were used as controls. Supernatants were harvested at the end of each experiment.

### Hematoxylin and eosin (H&E) and immunostaining

Fixed and embedded histological material was cut into 4-μm sections that were deparaffinized in xylene, rehydrated with graded alcohol and stained with H&E. Keratin and vimentin immunostaining was performed using an avidin-biotin-immunoperoxidase technique with the immunostaining kit, according to the manufacturer’s instructions.

### Quantitative PCR (qPCR)

Total RNA was isolated from the implanted endometrium using TRIzol reagent and, following the digestion of genomic DNA, was reverse-transcribed with the Real Time PCR Core kit. qPCR was performed on a Rotor-Gene 3000 real-time DNA analysis system (Corbett Research, Sydney, Australia) with the following primers: RANTES sense, 5′-ACCAGTGGCAAGTGCTCCAAC-3′ and anti-sense, 5′-CTCCCAAGCTAGGACAAGAGCAAG-3′; and GAPDH sense, 5′-GCACCGTCAAGGCTGAGAAC-3′ and anti-sense, 5′-TGGTGAAGACGCCAGTGGA-3′. PCR conditions were 95°C for 90 sec, 95°C for 5 sec, 60°C for 30 sec, 95°C for 1 min, 55°C for 1 min and 55°C for 10 sec, for 30 cycles. Relative quantification was performed using the cycle threshold (CT) 2^−ΔΔCT^ method, with relative quantification of the target calculated as: ΔΔCT = (CT_Target_-CT_GAPDH_)_X_ - (CT_Target_-CT_GAPDH_)_0_, where X is the treated group and 0 is the control group. Assays were conducted in triplicate for each sample.

### ELISA

The rhRANTES or recombinant mouse RANTES (rmRANTES; R&D Systems) levels in the peritoneal fluid or cell supernatants were determined using the previously mentioned ELISA kit, according to the manufacturer’s instructions. Peritoneal fluid was collected by irrigating the abdominal cavity with 1 ml cold phosphate-buffered saline (PBS) plus 0.02% ethylenediamine-tetraacetic acid. The samples were each centrifuged to remove cellular debris and then stored at −170°C.

### In vivo chemotaxis assay

Three mice were treated with 5 μM shikonin for 24 h and 2×10^7^ U937 cells were labeled with CFSE for 5 min prior to injection. The CFSE-labeled cells in 200 μl PBS were injected intravenously into the tail vein, while 10 μg rhRANTES in 200 μl PBS was injected intraperitoneally. Following 24 h, the peritoneal fluid was collected by irrigating the abdominal cavity with 7 ml cold PBS. Cells were harvested by centrifugation and CFSE-labeled cells were counted using a fluorescence microscope (IX71 Olympus; Tokyo, Japan).

### In vitro chemotaxis assay

Blood was obtained from the SCID mice by cardiac puncture and mixed with Hank’s solution in a 1:1 ratio. The mixture was resuspended in 2 ml mouse monocyte isolation buffer and centrifuged at 1,500 × g for 15 min to layer the mixture. The layer containing enriched monocytes was carefully removed and washed with Hank’s solution, followed by centrifugation at 2,000 × g for 10 min to collect the monocytes. Monocyte purity was >95% as determined by anti-CD14 staining in flow cytometric experiments.

Chemotaxis of the mouse monocytes in response to rmRANTES was assayed using 12-well chemotactic chambers (pore size, 5 μm; Costar, Cambridge, MA, USA). The upper chamber compartment was loaded with 100 μl mouse monocyte suspension (2×10^6^ cells/l). The lower compartment was filled with 600 μl RPMI-1640 with 2.5% FBS containing peritoneal fluid from the various groups of SCID mice, with or without rat anti-mouse RANTES (10 ng/ml; R&D Systems). Following 3 h at 37°C, the filters were stained with Giesma stain and the number of cells that had migrated to the lower surface of the filter was counted microscopically and was determined as the mean of 20 random high-power fields per well. Each assay was performed at least three times. The chemotactic index was the ratio of the number of cells that had migrated in response to chemokine, divided by the total number of cells.

### Statistical analysis

All data are expressed as mean ± SEM and were analyzed with the Statistical Package SPSS, version 12.0 for Windows (SPSS, Inc., Chicago, IL, USA). One-way analysis of variance was used to determine statistically significant differences among groups and the means of pairs of groups were compared using a Student’s t-test. P<0.05 was considered to indicate a statistically significant difference.

## Results

### Shikonin causes lesion regression

**Figure 1:**
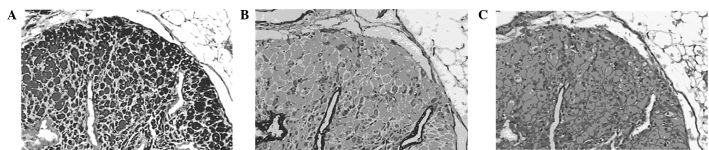
Identification of human/mouse chimeric models of endometriosis by immunostaining (magnification, ×100). Implanted endometrial lesions in the abdominal walls of SCID mice were stained for human (A) vimentin and (B) keratin. (C) Isotype control. SCID, severe combined immunodeficiency.

**Figure 2:**
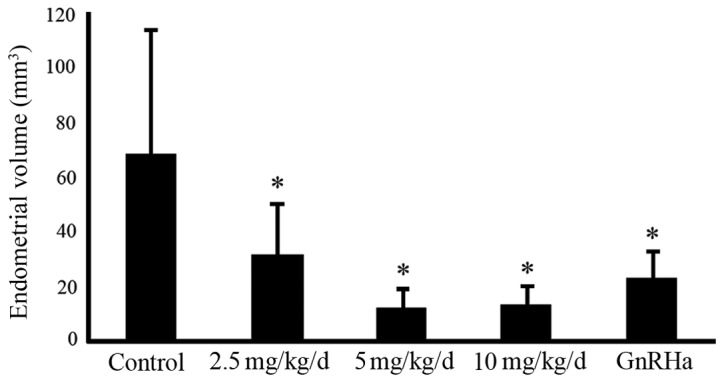
Shikonin inhibits lesion growth. Average lesion volumes from endometriosis model mice treated with shikonin at indicated doses or GnRHa (triptorelin) at 30 μg/kg. Error bars indicate SEM ^*^P<0.05, vs. normal/naïve control. GnRHa, gonadotropin-releasing hormone agonist.

Endometrial lesions in the model animals were verified by H&E staining and immunostaining, which showed endometrial acinar glands on a background of stromal cells. Glandular epithelial cells were cuboidal with clear cytoplasms and distinct vacuoles. The coexpression of keratin and vimentin, shown by immunostaining, indicated that endometriosis lesions had been established in the mice (Fig. 1). Compared with the lesions in the controls, those in the shikonin- and triptorelin-treated mice were significantly decreased in size. Although the high and intermediate doses of shikonin caused greater regression, no significant differences were observed between these two treatment groups (Fig. 2).

**Figure 3:**
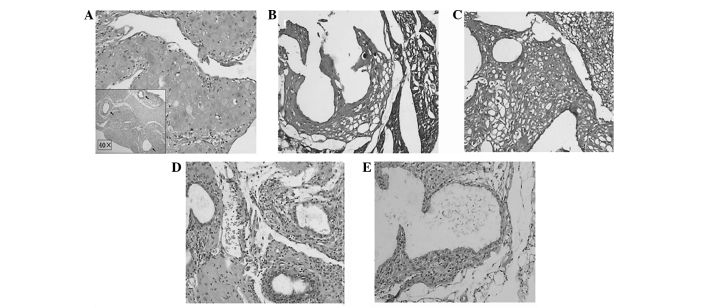
H&E staining of lesions transplanted into the peritoneal cavities of SCID mice. Mice were treated with shikonin at (A) a low dose, 2.5 mg/kg; (B) a intermediate dose, 5 mg/kg and (C) a high dose, 10 mg/kg. (D) Negative control and (E) triptorelin-treated positive control (magnification, ×400). H&E, hematoxylin and eosin; SCID, severe combined immunodeficiency.

In the groups treated with high and intermediate doses of shikonin, H&E staining showed marked atrophy of the endometrial acinar glands, scattered glandular epithelial cells, vacuolar degeneration of stromal cells, incomplete acinar structures and angionecrosis of adjacent vessels with glassy degeneration. Lesions in the triptorelin-treated group showed similar morphological changes. However in the low-dose shikonin group, endometrial tissues were a mixture of normal, atrophied and half-atrophied morphologies (Fig. 3).

### Effects of shikonin on RANTES expression

**Figure 4:**
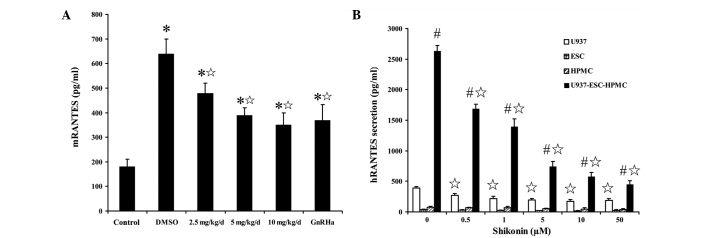
Effect of shikonin on RANTES expression. (A) mRANTES secretion in the peritoneal fluid of SCID mice following shikonin treatment at the indicated dose or GnRHa (triptorelin) at 30 μg/kg. (B) hRANTES secretion in U937, ESCs and HPMCs cultured alone or co-cultured, following shikonin treatment at the indicated dose, detected by ELISA. Data are presented as mean ± SEM. ^*^P<0.05, vs. normal/naïve control; ^¶^P<0.05, vs. DMSO groups; ^#^P<0.05, vs. groups cultured alone. RANTES, regulated upon activation normal T-cell expressed and secreted; SCID, severe combined immunodeficiency; ESC, endometrial stromal cells; HPMC, human peritoneal mesothelial cells; ELISA, enzyme-linked immunosorbent assay; GnRHa, gonadotropin-releasing hormone agonist.

The level of mRANTES secretion in the peritoneal fluid of the animal endometriosis model was markedly higher than that in normal SCID mice. However, this was not the case for hRANTES secretion. Following shikonin treatment, mRANTES secretion in the peritoneal fluid of the animal endometriosis model was decreased markedly in a dose-dependent manner (Fig. 4A). Human endometrium implanted in SCID mice also expressed low hRANTES at the transcriptional level.

Under normal conditions, U937 cells secreted more RANTES than HPMCs and ESCs and the co-culture of HPMCs with ESCs and U937 cells promoted significant RANTES secretion. However, following shikonin treatment, RANTES secretion by U937 cells and HPMC-ESC-U937 co-cultured cells was also significantly decreased compared with that in the controls (Fig. 4B). These observations indicate that shikonin may inhibit RANTES expression in the peritoneal cavity of females with endometriosis.

### Shikonin inhibits U937 cell chemotaxis in response to RANTES

**Figure 5:**
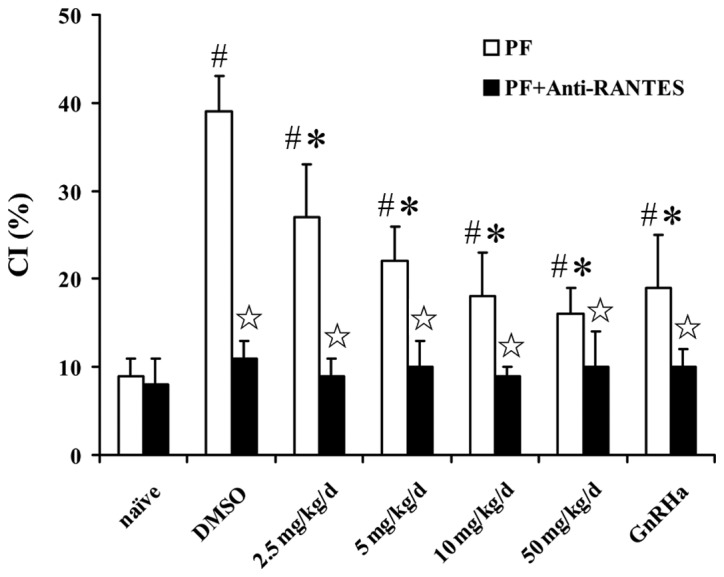
Shikonin (2.5, 5 and 10 mg/kg) reduces monocyte chemotaxis in the PF of SCID mice with endometriosis. Data are presented as mean ± SEM. ^#^P<0.05, vs. naïve control; ^*^P<0.05, vs. DMSO control; ^¶^P<0.05, vs. PF. PF, peritoneal fluid; SCID, severe combined immunodeficiency; RANTES, regulated upon activation normal T-cell expressed and secreted; GnRHA, gonadotropin-releasing hormone agonist.

**Figure 6:**
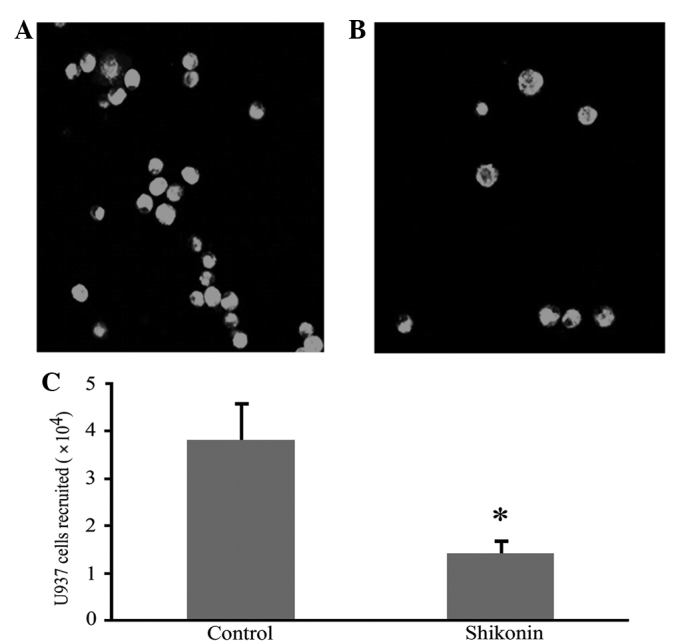
Shikonin inhibits U937 migration to the peritoneal cavity of SCID mice. Peritoneal fluid was collected from the (A) control and (B) shikonin-treated groups. (C) CFSE-stained cells were counted under a fluorescence microscope. Error bars indicate SEM. ^*^P<0.05 vs. normal/naïve control group. SCID, severe combined immunodeficiency; CFSE, carboxyfluorescein succinimidyl ester.

An *in vitro* chemotaxis assay showed that the peritoneal fluid from SCID mice with endometriosis promoted monocyte chemotaxis more than that of naïve mice. The chemotactic response was inhibited by shikonin in a concentration-dependent manner and a maximal inhibitory concentration of shikonin ranged between 10 and 50 ng/ml. The inhibitory effect was eradicated by adding mRANTES antibody to the peritoneal fluid (Fig. 5). Shikonin also significantly inhibited the migration of CFSE-labeled U937 cells to the peritoneal cavity *in vivo* (Fig. 6).

### Adverse effects and toxicity

No abnormal behavioral changes or clinical signs were observed in the mice at any of the doses of shikonin. In addition, no abnormalities were observed in the results of the serum biochemistry assays: ALT, AST, BUN, Cr, LDH, total protein, albumin and globulin data were within the normal ranges and no significant differences were identified between the results of the shikonin- and vehicle-treated groups.

## Discussion

To investigate the effects of shikonin on endometriosis, human/mouse chimeric models were established by injecting human endometrial tissue into the peritoneal cavity of SCID mice, which are widely used to study sensitivity to pharmacological therapy (10). H&E staining and immunohistochemistry assays demonstrated that ectopic implantation of human endometrium into the mice was accompanied by the formation of extensive adhesions of local peritoneal tissues. This further demonstrated that peritoneal inflammation was a significant characteristic of endometriosis (11).

In addition, the effects of shikonin on human/mouse chimeric SCID endometriosis were studied. It was shown that shikonin was capable of suppressing the growth of the implanted endometrium, decreasing the inflammatory responses induced by the graft and improving perilesional adhesions. In addition, shikonin exhibited significant inhibitory effects following administration at intermediate and high doses. Specific changes in H&E-stained specimens from intermediate- and high-dose treatment groups were observed, including significant atrophy of ectopic endometrial glands, sparseness of glandular epithelial cells (some of which had no integral structure), vacuolar degeneration of stromal cells and necrosis and atrophy of peripheral vessels accompanied by hyaline degeneration. However, no significant differences were found between the two groups. In the low-dose group, normal, atrophic and semi-atrophic states in the endometrial glands were all observed, with an even distribution of peripheral stromal cells, indicating a disparity in the sensitivity of glands to shikonin.

Endometriosis is an immuno-inflammatory disease characterized by peritoneal inflammation (11). Patients with endometriosis have a significantly increased number of macrophages compared with that in females without endometriosis, and their peritoneal fluid reveals abnormal expression of proinflammatory cytokines (12). As monocytes and HPMCs were obtained from mice and human-derived endometrium in the present experimental animal study, it was possible to detect the RANTES secretion from mice and humans. The results showed that the mRANTES secretion in the peritoneal fluid of the animal endometriosis model was higher than that in normal SCID mice and decreased markedly in a dose-dependent manner following shikonin treatment. By contrast, the expression of hRANTES by implanted human endometrial tissue was low at the transcriptional and translational levels.

As is commonly understood, the endometriotic tissue is composed mainly of the ectopic endometrium, peritoneal mesothelial cells, macrophages and extracellular matrix. It has been shown that there are increased levels of macrophages in the peritoneal cavity of females with endometriosis. These infiltrated macrophages are harbored in ectopic tissues and peritoneum (13). SCID mice, with combined deficiencies in T- and B-lymphocyte function, have a mononuclear macrophage system. Once human endometrium, as an exogenous antigen, is injected into SCID mice, it promotes the mononuclear macrophage system to induce immunological inflammation.

To further study the potential effect of shikonin on RANTES expression in a pelvic environment of endometriosis, a cell co-culture system was constructed. *In vitro* analysis of the expression of RANTES in U937 cells cultured alone or co-cultured with HPMCs and ESCs was performed. The results showed that U937 cells secrete more RANTES than HPMCs and ESCs. The co-culture of ESC-HPMC-U937 cells significantly promoted RANTES production and release. Shikonin showed a concentration-related inhibitory effect on the secretion of RANTES in U937 cells and ESC-HPMC-U937 cells. The results were also consistent with a previous study, which showed extremely low secretion levels of RANTES by *in vitro* cultured endometrial cells, whereas mononuclear macrophages cultured alone or co-cultured with HPMC were a significant source of RANTES secretion (9).

It is well known that RANTES is a potent chemokine for mononuclear macrophages (9). To further prove the potential role of shikonin on decreasing inflammatory responses, chemotaxis assays were performed. *In vitro* and *in vivo* chemotaxis assays demonstrated that shikonin had a significant effect on decreasing the sensitivity of monocytes to RANTES chemotactic signals.

In conclusion, the present study showed the potential ability of shikonin to inhibit the growth of ectopic endometrial tissue in human/mouse chimeric models. The mechanism of the therapeutic effect of shikonin may involve the inhibition of monocyte recruitment, and the downregulation of RANTES expression in the peritoneal cavity of females with endometriosis, followed by the alleviation of peritoneal inflammation. Further investigations of this compound may provide the basis for the development of new drugs for use in the treatment of endometriosis.
